# Caries and fluoridated water in two Brazilian municipalities with low prevalence of the disease

**DOI:** 10.11606/S1518-8787.2018052016330

**Published:** 2018-03-14

**Authors:** Mariângela Guanaes Bortolo da Cruz, Paulo Capel Narvai

**Affiliations:** I Universidade de São Paulo. Faculdade de Saúde Pública. São Paulo, SP, Brasil; II Universidade de São Paulo. Faculdade de Saúde Pública. Departamento de Política, Gestão e Saúde. São Paulo, SP, Brasil

**Keywords:** Fluoridation, Dental Caries, prevention & control, DMF Index, Oral Health, Cross-Sectional Studies, Fluoretação, Cárie Dentária, prevenção & controle, Índice CPO, Saúde Bucal Estudos Transversais

## Abstract

**OBJECTIVE:**

To analyze the association between exposure to fluoridated water and dental caries in a context of widespread use of fluoride toothpaste in Brazil, in a scenario of low prevalence of the disease.

**METHODS:**

This is a cross-sectional observational study, of the census type, in the form of a double population-based epidemiological survey carried out in two municipalities of the state of São Paulo in 2014. The sample consisted of adolescents aged 11 and 12 years, exposed (n = 184) or not exposed (n = 128) to fluoridated water for at least five years. The populations studied lived in communities of the same geographic region and had small demographic size and similar socioeconomic classification, differing only in the exposure (Silveiras) or not exposure (São José do Barreiro) to fluoridated water. The experience, magnitude, and degree of polarization of dental caries in these populations were analyzed using the DMFT and SiC indexes, and the association was tested using Pearson’s chi-square statistics and prevalence ratio between those not exposed and those exposed to fluoridated water.

**RESULTS:**

Although caries experience (DMFT ≥ 1) was not associated with exposure to fluoridated water (chi-square = 1.78; p = 0.18; α = 5%), a significant difference was observed in the magnitude with which the disease reached the population: the means of DMFT were 1.76 in those exposed and 2.60 in those not exposed and the means of SiC were 4.04 and 6.16, respectively. The degree of polarization, indicated by the percentage of subjects with DMFT = 0, was different, being it higher (41.8%) in subjects exposed and lower (34.3%) in subjects not exposed. The prevalence ratio between those not exposed and those exposed was 1.13, indicating little expressiveness in prevalence difference.

**CONCLUSIONS:**

Exposure to fluoridated water implied lower mean values for the DMFT and SiC indexes, even in the presence of the concomitant exposure to fluoridated toothpaste, in a scenario of low prevalence of the disease, and with a similar pattern of caries distribution in the populations analyzed.

## RESUMO

**OBJETIVO:**

Analisar a associação entre a exposição à água fluoretada e cárie dentária, em contexto de uso generalizado de creme dental fluoretado no Brasil, em cenário de baixa prevalência da doença.

**MÉTODOS:**

Realizado estudo observacional transversal, de tipo censitário, na modalidade de duplo inquérito epidemiológico populacional, em dois municípios paulistas em 2014. Participaram adolescentes de 11 e 12 anos, imersos na sua condição de exposição (n = 184) ou não exposição (n = 128) à água fluoretada, há pelo menos cinco anos. As populações avaliadas residiam em comunidades da mesma região geográfica, de pequeno porte demográfico e com classificação socioeconômica similar, diferenciando-se apenas pela condição de exposição à água fluoretada (Silveiras) e de não exposição (São José do Barreiro). A experiência, a magnitude e o grau de polarização da cárie dentária nessas populações foram analisadas por meio dos índices CPOD e SiC e a associação foi testada empregando-se as estatísticas qui-quadrado de Pearson e razão de prevalência entre não expostos e expostos à água fluoretada.

**RESULTADOS:**

Embora a experiência de cárie (CPOD ≥ 1) não tenha se associado com a exposição à água fluoretada (qui-quadrado = 1,78; p = 0,18; α = 5%), observou-se expressiva diferença na magnitude com que a doença atingiu a população: as médias CPOD foram de 1,76 em expostos e 2,60 em não expostos e as médias SiC foram de 4,04 e 6,16, respectivamente. O grau da polarização, indicado pela porcentagem de indivíduos com CPOD = 0, foi diferente, maior (41,8%) em expostos e menor (34,3%) em não expostos. A razão de prevalência entre não expostos e expostos foi de 1,13, indicando pouca expressividade na diferença das prevalências.

**CONCLUSÕES:**

A exposição à água fluoretada implicou menores valores médios dos índices CPOD e SiC, ainda que em presença de exposição concomitante a dentifrício fluoretado, em cenário de baixa prevalência da doença e padrão similar de distribuição de cárie nas populações analisadas.

## INTRODUCTION

There is no data to prove that public water fluoridation has preventive benefit to dental caries in addition to that provided by the widespread use of fluoride toothpaste in small demographic municipalities with low prevalence of the disease in Brazil. Dental caries is the number one oral health problem in the world, especially in developing countries^12^. The heterogeneous population distribution of the disease does not only arise from individual biological variations, from the “parasite-host” relationship, but, above all, from social differences that characterize persons and the context in which they are inserted^2^ ^,^ ^9^. Epidemiological studies that consider the concept of “social space”^6^, even though they are only descriptive, can contribute with data for public policies.

The Brazilian population is marked by deep socioeconomic inequalities and by iniquities in the distribution of caries. The unequal occurrence of the disease in different population groups generates situations in which few individuals accumulate the highest burden. This phenomenon is called “polarization of caries”, which is well characterized in national studies since the end of the last century^2^ ^,^ ^20^.

Considering the proved preventive action of fluoridated toothpastes, we have a justified interest in knowing whether the fluoridation of public water supply is still effective in contexts in which the use of these products is widespread, or whether it is neutralized or reduced to insignificant levels^15^. This justifies the evaluation of the preventive meaning of the exposure to fluoridated water for the control of the disease.

Fluoridated toothpastes, widely marketed in Brazil since the end of the 1980s, and water fluoridation by public supply systems, mandatory by law in the country since 1974, are the main preventive methods against dental caries^21^.

Water fluoridation is a Public Health technology that is basically the controlled addition of fluorine until a concentration considered as effective in preventing dental caries is reached. The preventive power of this measure depends on the maintenance and stability of the fluorine within appropriate levels^7^ ^,^ ^18^ ^,^ ^19^ ^,^ ^25^. It is considered a time-dependent technology, as exposure should happen at adequate concentrations, uninterruptedly, for at least five years for its maximum benefit^10^ ^,^ ^14^ ^,^ ^17^.

A systematic review of the beginning of this century with 214 studies on water fluoridation found that this method is effective for the prevention of dental caries. Although associated with very mild and mild levels of dental fluorosis, it is not associated with other adverse events^16^. It is estimated that its preventive power is approximately 40% to 70% in children, in addition to reducing tooth loss in adults from 40% to 60%^1^.

Antunes et al.^2^, using the Significant Caries Index (SiC), which reflects the impact of caries on the most affected individuals, in a sample of 18,718 students in São Paulo, have found values of 5.8 for those exposed to water fluoridation and 7.2 for those not exposed to it. The burden of disease in the region with the highest occurrence of caries was 24% lower among those benefited by fluoridation.

Brazil is the second country with absolute frequency in fluoridated water population coverage^17^. The Ministry of Health admits coverage of approximately 60% of the Brazilian population, with deep regional disparities^3^. In 2010, 75% of the United States population received fluoridated water. In 2009, in the state of São Paulo, Brazil, 93.5% of the population had fluoridation in the public network, distributed in 85.1% of its 645 municipalities. Among the municipalities of the state of São Paulo without fluoridation, 99% had less than 50,000 inhabitants^1^.

Even communities without fluoridation may have their populations indirectly benefited by fluoridated waters. Fluoride in the food and beverages produced in fluoridated areas is also ingested by the populations of these communities. This makes them relatively exposed and shows the diffuse mechanism of this method. This phenomenon is defined as the “halo effect”^8^ ^,^ ^20^.

Considering the scenario of the Brazilian macro-regions in the first decades of the twenty-first century, notably the South and Southeast, the low DMFT (mean number of decayed, missing, and filled permanent teeth) index, the polarization of caries, the halo effect, and the exposure to multiple fluoride sources, it is relevant to investigate whether exposure to fluoridated water has any benefit in caries prevention, especially in those most susceptible to the disease.

The objective of this study was to analyze the association between the magnitude of the dental caries experience in permanent teeth and the exposure to fluoridated public water in the context of widespread use of fluoride toothpaste.

## METHODS

This is a cross-sectional observational study, of the census type, in the form of a double, simultaneous, population-based epidemiological survey. We obtained the primary data in 2014 for the age group of 11 and 12 years, divided into those exposed (n = 184) and not exposure (n = 128) to fluoridated water for at least five years.

The preferred methodological design to analyze the association between dental caries and water fluoridation should include two social spaces^6^ that can be compared, with simultaneous data collection, compatible with the concept of community test. They should be matched by similar characteristics as social spaces, in which only exposure to public water fluoridation for at least five years could distinguish them. This methodological option is based on the recommendation of Barata and Werneck. For them, epidemiological studies that focus on geographical scenarios should broaden the physical concept of territory, understanding it, above all, as a “social space”, that is, social constructions resulting from the organized human action in society, acting on a certain landscape^6^. For this reason, we selected two municipalities that fully met the requirement of being similar “social spaces”, previously paired. Therefore, we could generate population parameters obtained from two simultaneous, directly comparable, censuses.

The first methodological procedure was the investigation of the history of fluoridation of all 645 municipalities of São Paulo in the National and State Systems of Information on the Surveillance of Drinking Water Quality in the period of seven years, from January 2008 to December 2014. This information for the two selected municipalities was checked based on laboratory reports (n = 128) of the water samples collected for health surveillance.

In order to measure compliance with the criterion of exposure or not to public water fluoridation, we adopted the requirement that at least 80% of the results of the analyses should be in agreement or in disagreement with Resolutions SS 250/1995^23^ and SS 65/2005^24^. These resolutions establish that the fluorine content in the water supply network in the state of São Paulo can range from 0.6 to 0.8 mg F/L, so that the population can be considered as benefiting from optimal levels.

Among the municipalities that met and did not meet the criteria of exposure to fluoridation and whose social spaces fulfilled the requirement of similarity adopted in this study, we identified the two most well-adjusted municipalities from the socioeconomic point of view, including cultural aspects: Silveiras (with fluoridation) and São José do Barreiro (without fluoridation). Both are located in the administrative region of Guaratinguetá, in the Paraíba Valley (Table 1).

**Table 1:** Socioeconomic characteristics of municipalities and quality of the public water fluoridation (Resolutions SS 250/1995 and SS 65/2005). Municipalities of São José do Barreiro and Silveiras and state of São Paulo, Brazil, 2010 to 2016.

Socioeconomic characteristics	Municipality		State of São Paulo

	São José do Barreiro	Silveiras	
Population in 2010 (a)	4,077	5,792	41,262,199
Population in 2015 (b)	4,185	6,158	44,396,484
Population in 2016 (c)	4,183	6,193	44,749,699
Percentage of population in rural areas, in 2015	31	51	4
MHDI and HDI, in 2010	0.684 (average)	0.678 (average)	0.783 (high)
Position in the São Paulo ranking of MHDI, in 2010 (n = 645)	617	625	-
IPRS, in 2012	5 (low)	5 (low)	-
Water supply - level of service by the public network in 2010 (%)	98.8	97.6	97.9
Number of laboratory tests performed (n = 128) from January 2008 to December 2014 and their respective percentage (%) of adequacy according to Resolutions SS 250/1995 and SS 65/2005	n = 44 (0%)	n = 84 (85.0%)	-

MHDI: Municipal Human Development Index; HDI: Human Development Index; IPRS: São Paulo Social Responsibility Index

Note: (a) 2010 IBGE Census; (b) 2015 IBGE Estimate; (c) 2016 IBGE Estimate.

Sources: 2016 IBGE; 2017 *Fundação* SEADE; 2017 PNUD; 2017 SABESP; 2015 National and State Water Quality Information Systems; and 2015 Instituto Adolfo Lutz de Taubaté.

The water collected for treatment and supply of the population comes from the Paraíba do Sul River Basin in both municipalities. In São José do Barreiro, the municipal sanitary system manager is the Municipal Water and Sewage Department (DAE) and in Silveiras, it is the Basic Sanitation Company of the State of São Paulo (SABESP).

The subsequent methodological step was to seek the proof of exposure and non-exposure of individuals, considering the mixed ecological study nature of the design. For this end, the map of the public supply network of Silveiras was obtained from SABESP. The entire urban area and much of the rural area received adequately treated fluoridated water, except for a small rural neighborhood (Sítio Bom Jesus), whose individuals were not part of this study. We also performed the mapping with the DAE of São José do Barreiro to prove that its waters were not fluoridated. In this way, we could classify individuals as exposed or not, applying the criterion of at least five years of residence in the same municipality and place. The admissibility of exposure was due to the characteristics of its compulsory nature in the places where fluoridation technology is used in the public water supply, and we cannot admit the non-exposure of individuals living in these social spaces.

The previous pairing, by similarity of social spaces of the exposed and not exposed populations, was assured by the use of two composite indexes: the Municipal Human Development Index (IDHM) and the São Paulo Social Responsibility Index (IPRS). The values of these indicators for each municipality allow us to identify social spaces with extremely similar characteristics (Table 1). In addition, this similarity is confirmed by the positions occupied by both places in the 2010 MHDI ranking of the state of São Paulo (n = 645), in which São José do Barreiro was in position 617 and Silveiras in 625 (Table 1).

Silveiras and São José do Barreiro are also similar in the environmental and historical aspects. They have an extensive territory with large rural areas, being located in the Paraíba Valley, Serra da Bocaina region, state of São Paulo, Brazil. Both were founded at the end of the eighteenth century, being old areas of coffee cultivation and passage of troops, and they became rich cities in the eighteenth and nineteenth centuries, with greater populations than the current ones. In the twentieth and twenty-first centuries, they lost economic relevance and became impoverished municipalities with economic and social dynamics in the small commerce, livestock, agriculture, and tourism.

The distance between Silveiras and São José do Barreiro is 47 km and no municipality “invades” them, which is quite common in large urban centers and their surrounding metropolitan areas.

Data collection was performed by four teams of examiners and annotators, being them dental surgeons and health aides. The time interval between the calibration of the teams and the field work was three days. The inter-examiner (K = 0.97–0.98) and intra-examiner agreements (K = 0.98–1.00) were considered optimal, which sows the internal and external consistency of the teams^25^.

Primary data collection was performed in school units, under good lighting and natural ventilation, and it was done simultaneously in both municipalities, in November 2014. We adopted the standardized criteria recommended by the World Health Organization (WHO) for epidemiological surveys in oral health^26^.

All educational establishments in both municipalities were included in the design, as we aimed to examine the entire population aged 11 and 12 years to analyze the permanent dentition. Education was only offered by public institutions in both municipalities in 2014. São José do Barreiro had six schools (two urban and four rural), while Silveiras had four schools (two urban and two rural).

According to the 2010 IBGE census, the dropout rate from four to seventeen years was approximately 10% in these municipalities. At the time of data collection, this information was conferred and ratified by the municipal and state education managers. Students begin to drop out gradually after the age fourteen, as some adolescents have to work to supplement the income of their families. The education managers also reported that the entire population aged eleven and twelve years was enrolled and routinely attending classes.

The teams made systematic returns to schools, according to the method, seeking to exhaust the possibilities of including all individuals. Losses were less than 5%, mainly resulting from the explicit or implicit refusal from parents or guardians and repeated absences from school. Thus, we set up the census aspect of this study.

Questionnaires were answered by parents or guardians and applied at the same time as the oral examinations, with the following variables: school identification, responsible guardian, examined individual, age, place and time of residence, source of water consumed, time of tooth brushing, toothpaste used, exposure to fluoridated mouthwashes at school, and brushing instruction.

We used the DMFT and SiC indexes. The DMFT index can be defined as the average number of decayed, missing, and filled permanent teeth at a given age, being the population located in space and time. It is considered a good indicator of oral conditions: the lower the DMFT index, the better the oral health situation of the individual examined^3^. However, in the current context of polarization and low prevalence of caries in Brazil, the DMFT index provides a sometimes incomplete view of the disease, especially in asymmetric distributions.

The Significant Caries Index (SiC), proposed by the WHO in 2000, may be an auxiliary epidemiological instrument to better characterize the distribution of caries in populations. It is calculated as the mean DMFT of a third of the individuals with the highest burden of disease (highest individual DMFT values). It thus translates the impact of caries on the most affected individuals and it is useful to describe scenarios of disease polarization^22^.

The reliability of the information on exposure was evidenced by the use of the triangulation technique. The following sources were used: a) the populations participating in the survey, b) the municipal managers responsible for education and health public policies (municipal secretaries, regional and state education representatives, school directors, and teachers), and c) the identification and analysis of official public documents. These sources were consulted for the knowledge on the presence of preventive oral health programs or strategies, such as fluoridated mouthwashes or supervised brushing, among others, which could have been performed at the same time as the period considered in this study. We performed this procedure because they could be characterized as preventive methods affecting the analyzed outcomes. We found that routine preventive activities were not carried out in both municipalities, at least in the last five years. The population studied, exposed and not exposed to fluoridated water, brushed their teeth, albeit irregularly, at least once a day with fluoride toothpaste and this was the only additional source of fluoride to which those individuals were exposed during the period considered in the study.

Data processing was performed using the EpiInfo software^11^. We evaluated the magnitude of the caries experience in the populations exposed (E) and not exposed (NE) to water fluoridation using the DMFT and SiC indexes. We measured the scaling of the difference in the prevalence of those not exposed and exposed using the prevalence ratio (PR) and we tested the exposure-disease association with Pearson’s chi-square test.

The research was approved by the Ethics Committee with CAAE process 34299614.7.0000.5421, 064944/2014, recognized by the CEP/CONEP system, respecting the requirements for this type of investigation^4^.

## RESULTS

The value of the DMFT index was 1.76 (SD = 1.92) for those exposed to fluoridated water and 2.60 (SD = 3.38) for those not exposed (Table 2).

**Table 2:** SiC and DMFT indexes and components for 11 and 12 years old, according to exposure to water fluoridation. Silveiras (with fluoridation) and São José do Barreiro (without fluoridation), state of São Paulo, Brazil, 2014.

Index	Exposure					

	Yes (n = 184)			No (n = 128)		
	
	n	Mean	%	n	Mean	%
SiC	63	4.0	100	43	6.2	100
DMFT	323	1.8	100	333	2,6	100
Decayed	89	0.5	27.6	287	2.2	86.2
Missing	7	0.1	2.1	7	0.1	2.1
Filled	227	1.2	70.3	39	0.3	11.7

SiC: Significant Caries Index; DMFT: Average number of decayed, missing, and filled permanent teeth

Mode was 0.0 in both distributions and the median was 1.0 for those exposed and 2.0 for those not exposed. The amplitude of variation was nine for those exposed and twenty-four for those not exposed. The coefficient of variation was 1.13 for those exposed and 1.30 for those not exposed.

**Figure 1:**
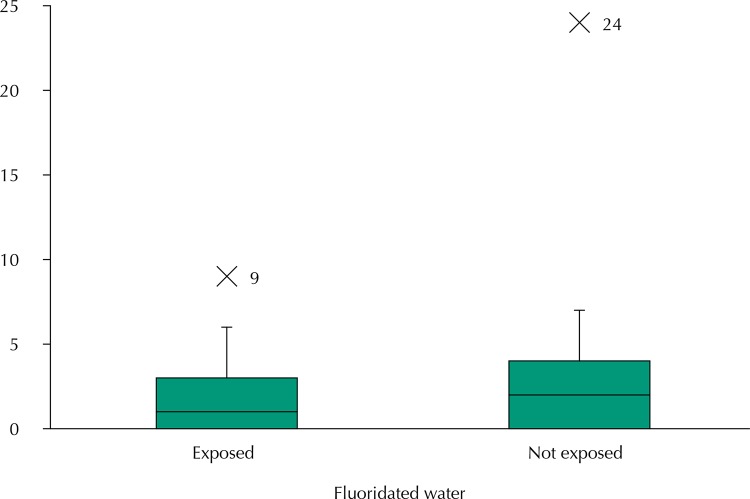
Box Plot of the distribution of the values of the DMFT index, in schoolchildren aged 11 and 12 years, according to exposure to fluoridated water. Silveiras (with fluoridation) and São José do Barreiro (without fluoridation), State of São Paulo, Brazil, 2014.

The box plot of Figure 1 shows the patterns of variability of the two populations, visually indicating the similarity of the dispersions and the central trends in them from the low prevalence of the disease. It shows that the different magnitudes represented by the values of the DMFT index were not from many aberrant values, since there was only one value with such characteristic in both distributions. In fact, the exclusion of aberrant values from the distributions minimally changed the means and standard deviations at irrelevant levels. The means changed from 1.76 to 1.66 among those exposed and from 2.60 to 2.43 among those not exposed. Differences in magnitudes were practically kept, decreasing by 1.3 percentage points: from 47.7% with full distributions to 46.4% for distributions without aberrant values. The respective standard deviations were 1.92 and 3.38 for the complete distributions and 1.84 and 2.81 for the distributions without the aberrant values. The differences in the standard deviations between the distributions of those exposed and not exposed were kept and were greater for those not exposed in both distributions with and without the aberrant values.

DMFT: average number of decayed, missing, and filled permanent teeth

**Figure 2:**
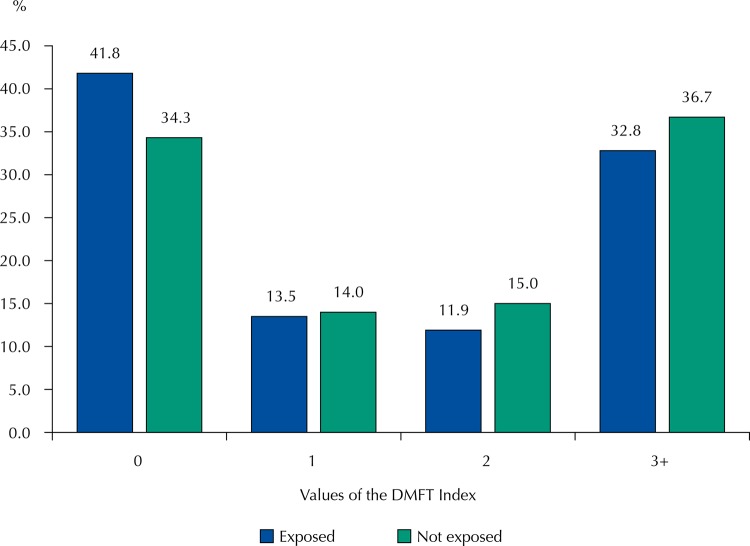
Percentage distribution of the values of the DMFT index, in schoolchildren aged 11 and 12 years, according to exposure to fluoridated water. Silveiras (with fluoridation) and São José do Barreiro (without fluoridation), state of São Paulo, Brazil, 2014.

The percentage of individuals without caries experience was 41.8% in those exposed and 34.3% in those not exposed (Figure 2).

DMFT: average number of decayed, missing, and filled permanent teeth

**Figure 3:**
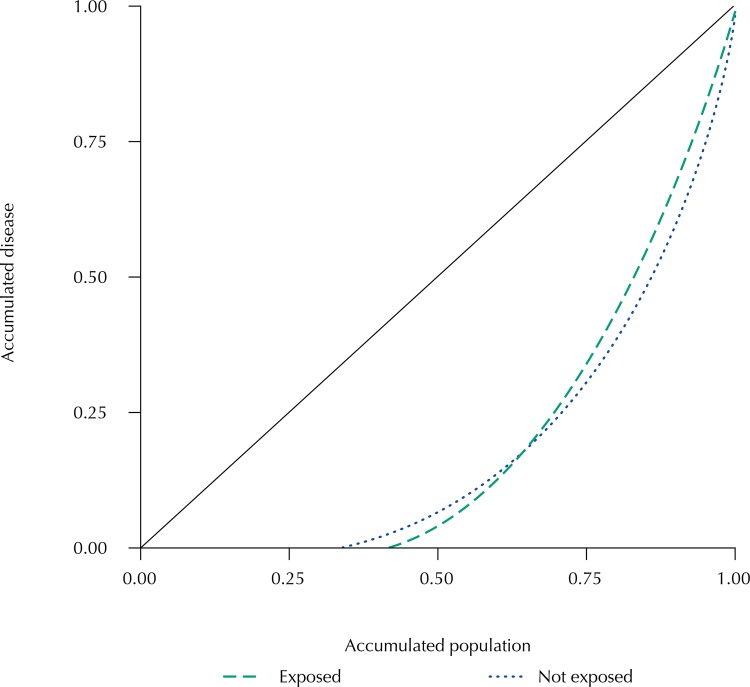
Lorenz curves for the load distributions of dental caries in schoolchildren aged 11 and 12 years, according to exposure to fluoridated water. Silveiras (exposed) and São José do Barreiro (not exposed), State of São Paulo, Brazil, 2014.

Both those exposed and not exposed presented distributions marked by moderate internal inequality (Figure 3). The Lorenz curves for those exposed and not exposed corresponded to Gini Coefficients of 0.5951 and 0.6158, respectively. We observed a marked similarity in the inequality patterns of both populations, showing that the different magnitudes represented by the DMFT index values were not from differences in the patterns of inequalities in the occurrence of caries in these populations.

The PR between those not exposed and those exposed (PR = NE/E) was 1.13, indicating little expressiveness in prevalence difference. The chi-square value (1.78; p = 0.18; α = 5%) indicated that the prevalence difference cannot be attributed to the exposure to the preventive measure.

## DISCUSSION

In epidemiological contexts with low prevalence of caries, in which the simple measurement of prevalence can obscure relevant differences in the degree to which the disease is expressed in individuals and populations, it is pertinent to inquire about the preventive impact of water fluoridation. One of the possibilities to evaluate this aspect is to analyze the magnitude of the DMFT and SiC indexes, since such epidemiological instruments allow us to measure this dimension beyond the traditional measures of incidence or prevalence. Among the requirements for such evaluations, from data obtained from cross-sectional studies, we can include ensuring the similarity of the socioeconomic conditions for comparison of the exposed and not exposed populations, as well as the absence of significant differences in prevalence. This research, the first one carried out in Brazil with these characteristics, consists of two epidemiological population censuses in populations differentiated only according to the exposure or not to public water fluoridation.

Cross-sectional studies have limitations. One is that they observe one moment in time and cannot, therefore, simultaneously measure the time of exposure to variables that may be associated with the observed outcomes. Such restrictions are applied to community assays when the goal is to analyze public health interventions with no records obtained before the cross-sectional moment. A methodological alternative to attenuate limitations of this type is represented by the strategy of studying concomitant moments in comparable populations, to a greater or lesser degree. This was the strategy adopted in this study^5^ ^,^ ^6^.

Although its specificities prevent the extrapolation of the results, this work was carried out in municipalities of small demographic size, whose populations are less than 10,000 inhabitants. These qualifications make up a study scenario that brings them closer to demographic conditions that are similar to those found in approximately half of the Brazilian municipalities, according to IBGE^13^. This gives the analysis some connotations that may be useful for decision makers who find themselves in similar situations. Cross-sectional studies are designs that can be very useful in supporting decision-making involving public policies^5^.

The population with no caries experience is 7.5 percentage points lower among those exposed than among those not exposed. On the other hand, in the area of highest burden of disease (DMFT ≥ 3), this difference is 3.9 percentage points greater among those not exposed to fluoridation. These values correspond to 21.9% more individuals with no caries experience among those exposed and 11.9% more individuals with a higher burden of disease (DMFT ≥ 3) among those not exposed. This data confirms that this preventive measure continues to produce relevant effects in the context of the study in the area of highest burden of disease, although the prevalence is not significantly different. There is a polarization in the distribution of DMFT in the populations exposed and not exposed to fluoridated water (Table 2 and Figure 2). Thus, the observed differences between these populations are even more relevant, since it is in an epidemiological context of low prevalence of caries, and yet the preventive effect provided by water fluoridation indicates that this measure is effective. The impact is more prominent in the area of highest burden of the disease.

The value (2.60) of the DMFT index obtained for those not exposed is 47.7% higher than the value (1.76) obtained for those benefited by water fluoridation, even though both populations were exposed to the fluorides in toothpaste in these municipalities. Both DMFT values are classified in the category of low prevalence by the WHO. This difference (47.7%), in the context of proved universal exposure to fluoride in toothpastes, represents original information of great value in the Brazilian context, marked by important health inequalities. It reveals the persistence of the preventive power of water fluoridation, although restricted to the scope of the study. This unprecedented finding suggests the relevance of the preventive potential of public water fluoridation in most Brazilian municipalities that do not have it, although we cannot say that it would reach the 48% found in this study. On the other hand, it is also relevant the impact on the values of the DMFT index when fluoridation is interrupted in municipalities that have been adopting this preventive measure. Such admissions result from the available knowledge about the Brazilian demographic and health reality and the systematic reviews on the efficacy of water fluoridation and it does not derive from the statistical inference of this particular study for the general situation of the country.

The SiC index in those exposed is 4.04, while it is 6.16 for those not exposed (Table 2). Non-exposure to fluoridated water increases the average number of DMF teeth by approximately 50% among adolescents, who concentrate the highest burden of disease. This value was more than twice that found by Antunes et al., in 1998, in which the difference was 24%^2^.

Public water fluoridation was identified as an environmental variable with the potential to explain the lower magnitude of the DMFT index among those exposed to the measure in the scenario of two small Brazilian municipalities, similar to most municipalities in the country in the first decades of the twenty-first century. This is compatible with the available theory and scientific evidence. In addition, in the area of highest burden of disease, even though it is in an epidemic scenario of low prevalence of the disease with the presence of daily use of fluoride toothpaste, the magnitude of the SiC index was approximately 50% lower for those exposed than for those not exposed to fluoridated water.
